# Long-term field studies in bat research: importance for basic and applied research questions in animal behavior

**DOI:** 10.1007/s00265-022-03180-y

**Published:** 2022-05-27

**Authors:** Gerald Kerth

**Affiliations:** Zoological Institute and Museum, Applied Zoology and Nature Conservation, University of Greifswald, Greifswald, Germany

**Keywords:** Bechstein’s bat, Climate Change, Conservation, Demography, Longevity, Social behavior

## Abstract

Animal species differ considerably in longevity. Among mammals, short-lived species such as shrews have a maximum lifespan of about a year, whereas long-lived species such as whales can live for more than two centuries. Because of their slow pace of life, long-lived species are typically of high conservation concern and of special scientific interest. This applies not only to large mammals such as whales, but also to small-sized bats and mole-rats. To understand the typically complex social behavior of long-lived mammals and protect their threatened populations, field studies that cover substantial parts of a species’ maximum lifespan are required. However, long-term field studies on mammals are an exception because the collection of individualized data requires considerable resources over long time periods in species where individuals can live for decades. Field studies that span decades do not fit well in the current career and funding regime in science. This is unfortunate, as the existing long-term studies on mammals yielded exciting insights into animal behavior and contributed data important for protecting their populations. Here, I present results of long-term field studies on the behavior, demography, and life history of bats, with a particular focus on my long-term studies on wild Bechstein’s bats. I show that long-term studies on individually marked populations are invaluable to understand the social system of bats, investigate the causes and consequences of their extraordinary longevity, and assess their responses to changing environments with the aim to efficiently protect these unique mammals in the face of anthropogenic global change.

## General introduction

In this review, I will address the significance of long-term field studies on bats for basic and applied research questions in animal behavior. For the general importance of long-term field studies in animals, I refer to the reviews by Clutton-Brock and Sheldon (2010) and Reinke et al. (2019). Notably, in a recent series of papers on long-term field studies in mammals (summarized in Hayes and Schradin 2017), bats have not been covered. The reason for this lack of coverage may be that long-term field studies on bats are rare, even more so than long-term field studies on other mammalian taxa. Being small, nocturnal and able to fly, bats present particular challenges to long-term research. At the same time, bats offer multiple reasons why long-term field studies investigating their behavior, demography, and population structure are highly rewarding. Bats are of high interest for research on aging (Foley et al. 2018; Wilkinson et al. 2021) and have diverse and sometimes complex social systems (McCracken and Wilkinson 2000; Kerth 2008; Wilkinson et al. 2019; Carter et al. 2020). They also provide multiple ecosystem services, including plant pollination, seed dispersal, and consumption of pest insects (Kunz et al. 2011). Finally, bats can carry viruses of high zoonotic potential such as Ebola, Marburg, or Corona viruses (Calisher et al. 2006; Letko et al. 2020; Mollentze and Streicker 2020), but little is known about virus transmission pathways within and between bat colonies (Zeus et al. 2020).

Bats have a life history that is quite different from that of other mammals: The vast majority of the more than 1400 bat species are group living, they are the only mammals capable of active flight, and most bat species use sophisticated echo-orientation systems, which allow them to navigate in complete darkness (Norberg and Rayner 1987; Kunz and Fenton 2007; Kerth 2008; Simmons and Cirranello 2020). Moreover, given their small size (*ca*. 3–1500 g), bats are extraordinary long-lived, with many species reaching a maximum age of 20 years and more (Barclay and Harder 2003; Munshi-South and Wilkinson 2010; Foley et al. 2018; Wilkinson et al. 2021).

Their peculiar life history makes bats very interesting for behavioral ecologists. At the same time, many bat species are of high conservation concern, with populations declining in most parts of the world (Mickleburgh et al. 2002; Racey and Entwistle 2003; Frick et al. 2020). In the face of the ongoing global biodiversity crisis, studying the behavior of bats can help to protect these extraordinary animals by addressing applied research questions and collecting conservation relevant data. The general relevance of studies on animal behavior for the protection of threatened species gave rise to the field of “Conservation Behavior” about 15 years ago (Buchholz 2007; Blumstein and Fernández-Juricic 2010).

One focus of this invited review will be on our long-term studies on Bechstein’s bats (*Myotis bechsteinii*), which we perform since 1993 with RFID-tagged and DNA-genotyped populations that live in deciduous forests in Southern Germany (Kerth and König 1996; Kerth and van Schaik 2012). But I will also report and discuss the results of other field studies on bats at the interface between conservation and behavior (Table 1). This includes two long-term studies that, like ours, run since several decades: One study investigates an English population of greater horseshoe bats (*Rhinolophus ferrumequinum*) for more than 60 years, which makes it the longest continuous study on a population of marked bats (Ransome 1989; Jones et al. 1995; Rossiter et al. 2005; Ward et al. 2014). The other study is on the critically endangered New Zealand long-tailed bat (*Chalinolobus tuberculatus*), living in temperate rain-forests in Fjordland, New Zealand, and which runs for three decades (Sedgeley and O'Donnell 1999; O’Donnell 2000; Pryde et al. 2005; O’Donnell et al. 2015).

**Table 1:** Overview of the long-term studies covered in this review. In these studies, bats have been individually marked, and their populations have been continuously monitored for at least 10 years (see text for more details). Colony/population sizes give an approximate range of the number of adult bats per colony/hibernation site. *N* of marked bats give a range of bats marked in each study, based on the given references or based on personal communication with the respective researchers

Species	Family	Region	Duration (years)	Study populations	Colony/population size	*N* marked bats	Main research topics	Main field methods	Genetic methods applied	Citizen scientists involved	References
*Chalinolobus tuberculatus*	Vespertilionidae	New Zealand/South Island	30	Several colonies in one large forest	ca. 150	> 4500	Conservation/demography/ffission-fusion dynamics/genetic population structure	Capture-mark-recapture/ringing/telemetry	Yes	Yes	Sedgeley and O'Donnell 1999/O’Donnell 2000/Pryde et al. 2005/O’Donnell et al. 2015
*Eptesicus fuscus*	Vespertilionidae	North America/Canada	15	One colony in one forest	40–50	> 100	Fission–fusion dynamics/roost choice/social thermoregulation	Capture-mark-recapture/ringing/RFID-tags/telemetry	Yes	No	Willis and Brigham 2004, 2007/Metheny et al. 2008/Bondo et al. 2019
*Myotis bechsteinii*	Vespertilionidae	Europe/Germany	30	Four colonies in four forests	10–50	> 900	Collective behavior/conservation/demography/fission–fusion dynamics/genetic population structure/parasites/social networks	Bat boxes/experiments/RFID-tags/telemetry	Yes	No	References provided throughout the text
*Myotis daubentonii*	Vespertilionidae	Europe/Germany	15	Three hibernation sites	> 2000	> 500	Conservation/demography/hibernation behavior	RFID-tags/capture-mark-recapture	Yes	Yes	Stumpf et al. 2017/Reusch et al. 2019/Meier et al. 2022
*Myotis daubentonii*	Vespertilionidae	Europe/England	20	Several colonies in one large forest	ca. 40	> 1500	Conservation/demography/phenology/roosting behavior/social structure	Bat boxes/ringing/capture-mark-recapture	No	Yes	Culina et al. 2017, 2019/Linton and Macdonald 2018, 2020
*Myotis nattereri*	Vespertilionidae	Europe/Germany	20	Several colonies and three hibernation sites	20–60 / > 2000	> 500	Conservation/demography/genetic population structure/hibernation behavior	Bat boxes/RFID-tags	Yes	Yes	Halczok et al. 2017/Stumpf et al. 2017/Reusch et al. 2019/Meier et al. 2022
*Myotis nattereri*	Vespertilionidae	Europe/Germany	12	One colony in one forest	ca. 80	> 300	Fission–fusion dynamics/genetic population structure/social networks/virus transmission	Bat boxes/RFID-tags	Yes	No	Halczok et al. 2017/Zeus et al. 2017, 2018, 2020
*Myotis nattereri*	Vespertilionidae	Europe/Germany	30	Several colonies in one large forest	20–60	3000	Demography/fission–fusion dynamics/genetic population structure	Bat boxes/capture-mark-recapture/ringing/RFID-tags	Yes	Yes	Halczok et al. 2017/Stapelfeldt et al. 2022
*Myotis nattereri*	Vespertilionidae	Europe/England	20	Several colonies in one large forest	ca. 40	> 500	Conservation/demography/phenology/roosting behavior/social structure	Bat boxes/ringing/capture-mark-recapture	No	Yes	Culina et al. 2017, 2019/Linton and Macdonald 2018, 2020
*Plecotus auritus*	Vespertilionidae	Europe/England	15	Several colonies in one forest	ca. 30	> 200	Conservation/demography/phenology/roosting behavior/social structure	Bat boxes/RFID-tags/experiments	No	Yes	Culina et al. 2017
*Plecotus auritus*	Vespertilionidae	Europe/Germany	20	Three colonies in one forest	10–20	> 200	Collective behavior/roosting behavior	Bat boxes/RFID-tags/experiments	Yes	No	Fleischmann and Kerth 2014/Zeus et al. 2017
*Rhinolophus ferrumequinum*	Rhinolophidae	Europe / England	60	One colony in an attic and several hibernation sites	ca. 200	14000	Aging/conservation/demography/genetic population structure/mating behavior	Capture-mark-recapture/ringing	Yes	Yes	Ransome 1989, 1995/Ransome and McOwat 1994/Jones et al. 1995/Rossiter et al. 2000, 2001, 2005/Ward et al. 2014/Foley et al. 2018

## What do I mean, when I speak of a long-term field study in bats?

The definition of a long-term field study depends on the study organism and the research question tackled (Reinke et al. 2019). Reinke and co-workers state “a key aspect of what makes a study long-term is the ability to observe and understand temporal variation.” For example, if occasional disease outbreaks or rare catastrophic weather conditions shape the population dynamics of a long-lived species, even a study covering 20 years may not allow enough replicates to identify the factors causing population crashes. Thus, in the context of responses to climate change or rare catastrophic events, a study should probably cover more than two decades in order to be truly termed “long-term.” In contrast, a 10- to 15-year study on the social behavior of long-lived species typically will allow to unravel the dynamics of the social structure and to document even rare behaviors such as occasional dispersal events in a highly philopatric species.

For the remainder of this paper, I will refer to “long-term” field studies if they cover at least three generations or twice the average lifespan (= life expectancy) of the population under study. For most bats, that means that a field study needs to cover at least 10–15 years to justify it being called “long-term” (for comparison*:* the life expectancy for Bechstein’s bats is about 5–6 years, Fleischer et al. 2017; generation time is about 4–5 years, Mundinger et al. 2022). My rationale for this definition is that it only becomes possible to measure the life-time reproductive success of individuals and the temporal variation in population dynamics and social structure if a study covers at least such a time frame. Moreover, I will only address long-term field studies where bats have been individually marked, and most marked individuals have been observed/recaptured many times during the study period. Thus, I will not discuss the results of large-scale banding studies on bat migration, which sometimes span several decades but where recapture rates are typically very low (< 5%) and where the bats often cannot be assigned to a specific population (Steffens et al. 2007). In large-scale banding-studies of this kind, individuals are recaptured on too few occasions to study the causes and consequences of behaviors at the individual level. Table 1 provides an overview of long-term studies on bats I am aware of.

## Why do we need long-term field studies on bats?

Due to the longevity of bats, certain research topics such as changes in the phenology, life history, or social systems in response to environmental change can only be addressed with individualized long-term field data (compare Clutton-Brock and Sheldon 2010). However, it is important to note that many other interesting research topics do not require long-term field studies. Examples include studies on the echo-orientation of wild bats, which can be carried out during one or two field trips (e.g., von Helversen and von Helversen 1999; Siemers and Schnitzler 2004; Schöner et al. 2015). Studies using population genetic tools to investigate cryptic behavior such as dispersal or mate choice typically require field work of maximally a few years to obtain the required DNA samples (e.g., Petit and Mayer 1999; Burland et al. 2001; Rivers et al. 2005; Dechmann et al. 2007). Moreover, experimental field studies that cover a few seasons can reveal fascinating insights into the behavior of wild bats. This includes studies on communication and cognition in bats (e.g., Page and Ryan 2005; Hernández-Montero et al. 2020; Fernandez et al. 2021). Finally, even field studies addressing the effect of anthropogenic changes of the environment on bats, such as the impact of light pollution on habitat use, do not always require individualized long-term data (e.g., Stone et al. 2009).

Long-term individualized field studies are, however, essential whenever measuring survival and lifetime reproductive success of individual bats is required for addressing a research question. Lifetime reproductive success (total progeny produced by an individual) is a well-established but difficult to obtain measure of individual fitness in wild mammals (Clutton-Brock 1988). Information on individual fitness is required for evaluating the ultimate causes of behavior as well as for understanding the drivers of population dynamics, which is crucial for conservation (Clutton-Brock and Sheldon 2010). Because of their longevity and low annual reproductive output, measuring lifetime reproductive success in a number of bats large enough to allow for statistical analyses will often require the monitoring of dozens of individuals for a decade or more (*e.g*., Ransome 1995; Ward et al. 2014; Culina et al. 2019; Mundinger et al. 2022). Finally, to understand the factors shaping the dynamics of wild bat populations in response to changing environments, such as global warming or predation by introduced predators, long-term data are typically needed (Pryde et al. 2005; Culina et al. 2017; Fleischer et al. 2017; Linton and Macdonald 2018, 2020; Mundinger et al. 2021, 2022).

## What are the challenges of long-term field studies on bats?

Researchers working on free-ranging bats face numerous challenges, some of which are multiplied if one attempts to work on the same population for a long time. The nocturnal activity of bats, in combination with the fact that many species spend the day hidden in often inaccessible roosts (Kunz and Fenton 2007), often requires the use of technical devices such as radio- and RFID-tags or infra-red video-recording that allow for monitoring the behavior of bats in roosts without disturbing the animals (Kunz and Parsons 2009). Further obstacles to the study of bats are the fact that their vocalizations are typically in the range of ultrasound, which again requires special devices to study their orientation and communication system (Kunz and Parsons 2009). Finally, because bats can fly, their home-ranges, dispersal distances, or seasonal migrations often exceed the detection range of small bio-logging devices such as radio-telemetry-transmitters. At the same time, most bat species are too small to carry GPS-tags, which if applicable are a fantastic tool to monitor the movements of bats over large distances (e.g., Harten et al. 2020). Current recommendations are that tag-weight should not exceed 10% of the bats’ body mass and be less than 5% if bats are tagged for longer than just a few days (O’Mara et al. 2014). This means that for most bat species, tag-weight should not exceed 1 g. In this context, it is important to emphasize that in Europe and many other countries, bats are strictly protected by law (Barova Streit 2018). In addition to the permits for working with protected species, animal welfare permits are required in many countries for marking bats with RFID-tags or other bio-logging tags. The same applies for taking wing-tissue samples for assessing the genetic relatedness among the study animals. The need to obtain permits for working with animals is of course not restricted to bats, but their small size limits what kind of techniques are applicable without interfering with their wellbeing.

To overcome the difficulties of directly observing the behavior of bats, population genetic tools can be used to address research questions dealing with dispersal, gene flow, kin selection, mating behavior, reproductive success, and seasonal migrations (e.g., Petit and Mayer 1999; Kerth et al. 2000; Rossiter et al. 2000, 2005; Burland et al. 2001; Rivers et al. 2005; Ward et al. 2014). For example, as direct observations of mating events and lactation events of marked individuals are difficult to obtain in wild bats, population genetic tools are typically needed for constructing pedigrees, which are relevant for quantifying fitness-related parameters and individual traits. As a consequence of the difficulties of obtaining both, detailed demographic data and comprehensive DNA-samples from wild bat populations, multi-generational pedigree data are available for only a few bat species (e.g., Kerth et al. 2002b; Rossiter et al. 2005; Nagy et al. 2007). While being scientifically highly rewarding, the combination of technology-driven monitoring and population genetic tools makes field studies on bats often cost-intensive. For long-term field studies this is even more relevant, as these costs must be covered over long time periods. Clearly, this does go well with the early phase of an academic career and the typically short-term funding regime in science. Consequently, most long-term studies were not intended to become “long-term” when they were initiated by early-career researchers. Another inherent challenge to long-term field studies is that it is often logistically difficult to intensively monitor more than one study population, which makes it very hard to achieve replicates (compare Table 1).

The fact that long-term studies typically develop from studies that had originally been designed to address a specific research question causes problems of its own. Most importantly, the consistency of data collection and long-term data storage is not always guaranteed over the course of an “emerging” long-term study, e.g., due to the almost inevitable personnel turnover. Maintaining standardized data collection is difficult when it is passed on every few years, for example, to a new cohort of doctoral students. Protocols that describe how to collect and store field data can certainly facilitate standardization, but some variance in data collection is unavoidable in long-term field studies. For example, field experiments carried out to address certain research questions can complicate the analyses of long-term data on other research questions if the experiments interfere with the standard data collection or the setup of field sites. Some of these issues may be resolved by a careful data cleaning and the use of modern statistical tools such as generalized linear mixed models (GLMMs) that can deal with noisy and heterogeneous data (Harrison et al. 2018). Nevertheless, data heterogeneity remains a major challenge for long-term studies.

## How do current methodological advances facilitate long-term field studies on bats?

The study of bat behavior has profited enormously from the breath-taking range and speed of technological advancement over the last decades. One striking example is the automatic monitoring of bat roosts with RFID-tag loggers. Implanted RFID-tags have first been used to mark bats in the 1990s (Kerth and König 1996; Brooke 1997) and are now a well-established method for the automatic monitoring of bat roosts (Kerth and Reckardt 2003; Patriquin et al. 2010; Kerth et al. 2011; Burns and Broders 2015; Reusch et al. 2019; van Harten et al. 2019). The biggest advantage of this method — compared to classical banding studies that rely on the capture-mark-recapture of animals — is that it allows to document an individual’s arrival and emergence without interfering with the animals at the roost. However, this works only if the roost entrance is small enough for placing an antenna (typically in the range of less than 0.5 m^2^). Moreover, the automatic identification of individuals passing through the antenna is most accurate if bats crawl instead of flying through. This is probably the reason why many long-term field-studies on bats that used the RFID-technology work with species that roost in tree cavities and bat boxes (Table 1).

In more recent years, novel bio-logging technologies, such as proximity tags, enable bat researchers to monitor the social behavior of bats even outside of their roosts (Ripperger et al. 2020). The miniaturization of bio-logging tags nowadays allows for documenting behavior even in small bat species. However, while passive induced RFID-tags allow monitoring the behavior of bats over their entire lifespan (Kerth et al. 2011), radio-transmitters, GPS-tags, and proximity tags require batteries and thus can only be fitted to bats for a limited time. At the same time, more traditional behavioral observation methods of bats, such as infrared video-monitoring and thermography, continue to be important (Kunz and Parsons 2009). Overall, no single method will allow to address all research questions arising during a long-term study. Thus, a careful evaluation of the advantages and disadvantages of each method for the particular research question and study species is always required.

## What did we learn from three decades of studying wild Bechstein’s bats?

Information on whether or not individuals disperse from their natal area or their social group is essential for understanding the social systems of animals (e.g., Clutton-Brock 2016) and for protecting their populations. In bats of the temperate zone, population genetic studies as well as capture-mark-recapture studies revealed that females are typically philopatric to their natal colony (e.g., Petit and Mayer 1999; Burland et al. 2001; Castella et al. 2001; Metheny et al. 2008). Female Bechstein’s bats take this to the extreme: During 25 years of intense roost-monitoring with RFID-loggers in combination with genetic mother–offspring assignment in four colonies (Kerth and van Schaik 2012), only two females’ immigration events were documented. Population genetic studies of a large number of colonies, living in Germany and Bulgaria, confirmed that females are highly philopatric, independently from the region where their colonies are living (Kerth et al. 2000, 2002b, 2008; Kerth and van Schaik 2012).

Why are Bechstein’s bat colonies closed societies? In our study sites, no ecological barriers exist that would restrict females from moving between the monitored colonies. Adult males, who live solitarily during summer, always disperse from their natal colony and often settle in the area of another maternity colony (Kerth et al. 2002a; Kerth and Morf 2004). Moreover, using confrontation tests, we could show that females can discriminate between members of their own colony and females that belong to foreign colonies. While females showed no aggression toward colony mates, they attacked foreign females that entered their roost during our confrontation tests (Kerth et al. 2002b). This suggests that colony members prevent the immigration of foreign females at times. The recognition of foreign females is probably based on olfactory cues and possibly also on vocal cues (Safi and Kerth 2003; Siemers and Kerth 2006). However, in multiple years of nightly infra-red video-recording in day roosts (e.g., Kerth et al. 2003a), we never observed aggressive behavior indicative of an immigration attempt. Thus, even unsuccessful immigration attempts seem to be rare.

Why should colony members prevent foreign females from entering their day roost? In many mammal societies, group members show some degree of territoriality and xenophobic behavior (Clutton-Brock 2016). The reason for aggression toward foreigners is typically the defense of crucial resources, such as feeding areas or mates, as found in meerkats (*Suricata suricatta;* Bateman et al. 2015). In Bechstein’s bats, however, it is unclear which critical resources a colony could defend. Unlike in many other bat species (McCracken and Wilkinson 2000), mate defense plays no role, as there are no adult males present in the colonies and mating takes place apart from the summer habitat (Kerth et al. 2003b; Kerth and Morf 2004). It is also unlikely that colony members are able to defend their foraging sites by defending communal day roosts. Colony members forage in individual areas that are widespread in the forest and are typically located several hundred meters from the colony’s day roosts (Kerth et al. 2001a; Kerth and Melber 2009; Melber et al. 2013).

Do colonies defend their roosts because suitable roosts themselves are a limited resource? In one of our study sites, Natterer’s bats (*Myotis nattereri*) preferentially occupy roosts recently used by brown-long eared bats (*Plecotus auritus*) but not by Bechstein’s bats (Zeus et al. 2017). This suggests there is some competition about day roosts between forest-living bat species. However, female Bechstein’s bats use up to 50 communal roosts (bat boxes and tree cavities) during their 5-month breeding season (Kerth and König 1999; Kerth et al. 2011). Moreover, during their almost daily roost-switching, colonies often split into subgroups that use separate day roosts before they mix or fuse again (Kerth and König 1999; Kerth et al. 2011). Fission–fusion behavior and frequent switching of roosts is widespread in forest-living bats (Kerth and König 1999; O’Donnell 2000; Willis and Brigham 2004; Popa-Lisseanu et al. 2008; Zeus et al. 2017). For colonies that switch roosts every other day, a single roost may be of limited value and probably can only be defended when the colony occupies it.

Are there costs to accepting foreigners into colonies? Energetic benefits from social thermoregulation are often used to explain why female bats form colonies to communally raise their offspring (Willis and Brigham 2007; Kerth 2008). However, social thermoregulation is unlikely to be the driver for xenophobic behavior in Bechstein’s bats. For social thermoregulation to be most efficient, the number of bats that cluster in a roost should be more important than the individual composition of a group (Pretzlaff et al. 2010; Küpper et al. 2016). Thus, it seems unlikely that by accepting a foreign female, colonies would impair their social thermoregulation capabilities. A similar argument may apply to two other social behaviors of female Bechstein’s bats that ensure the functioning of their colonies: information transfer and collective decision-making about communal day roosts (Kerth and Reckardt 2003; Kerth et al. 2006; Fleischmann et al. 2013). It remains unclear whether the immigration of foreign females would compromise the integrity of the fission–fusion society of female Bechstein’s bats, which strongly depends on a coordinated roost-switching. It seems likely that immigrants at first are unfamiliar with the local area. Thus, they may initially contribute less to the information transfer about communal roosts than the original colony members. But the same applies to the young females born in the colony, which also first need to learn about the roosts in the colonies home range but which are nevertheless allowed to stay in the natal colony. Female Bechstein’s bats are egalitarian breeders with low reproductive skew (Kerth et al. 2002a). Thus, eviction of females in order to avoid reproductive competition, a behavior which occurs in mammal species with a strong female reproductive skew (Clutton-Brock 2016), also does not apply to Bechstein’s bats. Finally, there is mixed evidence for kin selection in Bechstein’s bats (Kerth and Reckardt 2003; Kerth et al. 2011). Because the females mate promiscuously with males born in foreign colonies, colonies consist of closely related as well as genetically largely un-related females. This leads to an overall low average relatedness in colonies despite strong female philopatry (Kerth et al. 2002b). In summary, neither reproductive competition among females nor resource defense or kin-based cooperative behavior may explain why female Bechstein’s bats live in closed societies.

Could parasite avoidance be the reason for living in closed societies? By avoiding contact with foreign females, Bechstein’s bats could limit the introduction of parasites into their colonies. Bats carry a variety of parasites and viruses, many of which are contact transmitted and thus could possibly be avoided by living in closed societies (Kerth and van Schaik 2012). Overall, surprisingly little is known which fitness costs the different parasites and pathogens inflict on their bat hosts under natural conditions, with the most notable exception of the fungus *Pseudogymnoascus destructans*, causing mass mortality in North-American bats (e.g., Frick et al. 2020). For Bechstein’s bats, we have no direct information on the costs of carrying blood-sucking wing mites (*Spinturnix bechsteini*) and bat flies (*Basilia nana*), both of which are largely host-specific. However, we assume such costs exist, as it is known that wing mites can cause energetic costs in mouse-eared bats (*Myotis myotis*; Giorgi et al. 2001) and because female Bechstein’s bats show several behaviors that help them to reduce parasite load. For example, females allo-groom each other (Kerth et al. 2003a), a behavior also observed in other bat species (Carter and Leffer 2015). Moreover, by regularly switching day roosts and by avoiding previously occupied roosts, Bechstein’s bats can reduce the infestation with bat flies whose contagious puparia remain in the roosts after the bats moved on (Reckardt and Kerth 2007).

Population genetic studies showed that Bechstein’s bat colonies harbor wing mites that are genetically differentiated from those occurring in neighboring colonies (Bruyndonckx et al. 2009; van Schaik et al. 2014). This suggests that the purely contact-transmitted mites are not exchanged between colonies during summer when the bats live in closed societies. However, this does not prevent parasite transmission during other periods of the bats’ yearly life cycle. By comparing the genetic composition of mite populations of the same Bechstein’s bat colonies between years, we could show that there is a large genetic temporal turnover in the mite population of a given bat colony (Bruyndonckx et al. 2009). Bechstein’s bats, as many other bats of the temperate zone, mate in autumn at “swarming sites,” where males and females from many different colonies meet (Kerth et al. 2003b; Rivers et al. 2005). There, the bats cannot avoid contact to non-colony members, e.g., males that may function as vectors, and this apparently leads to the transfer of parasites between colonies that have no contact during summer. Thus, living in a closed society during summer does not prevent the spread of contact-transmitted parasites during the autumn mating season. Moreover, although the peculiar social system of Bechstein’s bat has an impact on their wing mites and bat flies, parasite life history, such as transmission mode and the mortality toll during winter, is also a major player in the system (van Schaik et al. 2015). Thus, to make 30-year research story short: we still do not fully understand why Bechstein’s bat colonies are closed societies.

Although we cannot explain why female Bechstein’s bats stay in their natal colony throughout their life, the implications for conservation management are clear: each colony needs to be protected as a demographically independent unit (Kerth et al. 2000). At the same time, gene flow must be maintained by protecting central mating sites (Kerth et al. 2003b). Moreover, the limited dispersal and colony foundation capacities of the species (Kerth and Petit 2005; Kerth and van Schaik 2012) requires that conservation management needs to enable populations to cope with climate change in situ. This first requires that we know which factors shape the local population dynamics of Bechstein’s bats. In a recent paper, Mundinger et al. (2021) used long-term data to show that juvenile Bechstein’s bats born in warmer summers grew to larger body sizes. With an increasing number of warmer summers over the last two decades, juveniles thus grew larger on average. While this is interesting per se, as many species shrink in response to global warming (Sheridan and Bickford 2011), growing larger has also negative consequences for the survival of females. In our study population, larger females have a higher mortality risk (Fleischer et al. 2017; Mundinger et al. 2021). Thus, with ongoing climate change, populations may decline if females continue to grow to large sizes and their higher mortality is not offset by a higher reproduction. Interestingly, there is evidence that larger females indeed show a faster pace of life, reproducing at an earlier age than smaller females (Mundiger et al. 2022). For long-term population persistence, however, mitigating the negative effects of global warming may require an improved local habitat quality with a high food availability and a large number of tree cavities that offer a wide range of micro-climatic conditions (compare Kerth et al. 2001b).

## Future perspectives of long-term field studies on bats

With the ongoing anthropogenic global change, long-term studies that investigate the responses of bats to changing environments are more important than ever. In birds, long-term monitoring data revealed shifts in the timing of reproduction and the phenology of seasonal migrations, raising concerns about an increasing mismatch between prey availability and the species’ breeding seasons (Cotton 2003; Hällfors et al. 2020). In contrast, relatively little is known about shifts in the breeding or hibernation phenology of bats in response to global warming (but see Linton and Macdonald 2018, 2020; Mundinger et al. 2021; Meier et al. 2022; Stapelfeldt et al. 2022). Moreover, a few studies used climate-envelope models to predict the future distribution of bat species (e.g., Razgour et al. 2013). However, such studies often suffer from the lack of data on the capability of bats to cope with changing environments in situ, e.g., by adjusting roost selection or by switching to alternative prey. Clearly, more long-term studies that measure the flexibility of individual behavior of bats are needed to assess whether bats can successfully adapt their hibernation and breeding phenology to changing climatic conditions.

In a recent study where we analyzed our long-term monitoring data, we were able to show that rare population crashes drive the population dynamics in Bechstein’s bats (Fleischer et al. 2017). However, it remained unclear what exactly caused the observed increase in mortality in such catastrophic years. We assume that cold weather conditions in autumn when the bats need to accumulate fat for hibernation and in spring at the time when bats emerged from the hibernacula caused the observed high mortality. At the same time, there is evidence from other long-term studies on bats that weather conditions have a species-, age- and sex-specific effect on bats (Culina et al. 2017; Linton and Macdonald 2018; Reusch et al. 2019; Stapelfeldt et al. 2022). All this makes predictions on population persistence in bats even more difficult and warrants for more long-term field studies on additional bat species and in different regions of the world.

Overall, basic questions relating to the demography of bats are still largely unanswered: For example, to what degree do intrinsic and extrinsic factors influence mortality risk and reproductive success in bats, and how do both factors interact with each other. The available long-term data suggest that demographic (age), genetic (heterozygosity), morphology (size), and environmental conditions (weather; predation) all affect mortality in bats (Rossiter et al. 2001; Pryde et al. 2005; Schorcht et al. 2009; Ward et al. 2014; Culina et al. 2017, 2019; Reusch et al. 2019; Mundinger et al. 2021). However, without individualized long-term data on further species, including bats of the tropics, for which almost no such data exist, an efficient conservation management of bat populations at times of a rapidly changing world is difficult. The long-term study on the New Zealand long-tailed bat by O’Donnell and co-workers (Sedgeley and O'Donnell 1999; O’Donnell 2000; Pryde et al. 2005) is a prime example how long-term capture-mark-recapture data, combined with behavioral and environmental data, can help to protect endangered bat populations: In the New Zealand long-tailed bat, increased predation by introduced predators in masting years of southern beeches (*Nothofagus* spec.) reduces the bats’ survival probability and leads to re-occurring population crashes that ultimately will drive the species to extinction (Pryde et al. 2005). The authors showed that only a significant reduction of the predation pressure though predator control can save the New Zealand long-tailed bat in the long term.

Being exceptionally long-lived, bats are of high interest to researchers studying aging. But even studies that entirely focus on the molecular mechanisms underlying the extraordinary longevity of bats require long-term field data from marked individuals to collect the relevant samples from bats of known (old) ages (Foley et al. 2018; Wilkinson et al. 2021). Moreover, in order to understand the ultimate and proximate causes of bat longevity and the reasons for their negligible senescence (Fleischer et al. 2017; Foley et al. 2018), long-term field studies are needed that investigate how reproduction decisions influence individual mortality risks and ultimately individual fitness (Ransome 1995; Culina et al. 2019; Mundinger et al. 2022).

The current SARS-CoV-19 pandemic is a fresh reminder that outbreaks of zoonotic viruses are sometimes connected to bats, even though bats are not exceptional in how may zoonotic viruses they carry compared to other mammals after accounting for species richness (Mollentze and Streicker 2020). However, if we want to go beyond a mere description of viruses occurring in bat populations, we need long-term studies that monitor virus dynamics in individually marked bat populations and relate the detected virus load to the individual behavior of bats such as forming close individual roosting associations (e.g., Zeus et al. 2020).

One novel and highly rewarding aspect of field studies on bats is the establishment of captive colonies that later are re-introduced into the wild, where they can be investigated for a long time. Such an approach has been highly successfully in case of the “in-house” colony of wild Egyptian fruit bats (*Rousettus aegyptiacus*) established by Yovel and co-workers (e.g., Harten et al. 2020). A similar approach has been carried out in vampire bats (*Desmodus rotundus*) in Panama, again leading to fascinating insights into bat social behavior (Carter et al. 2020). Unfortunately, the establishment of captive colonies is only possible in species such as in fruit-eating bats where feeding bats in captivity is feasible. In most insectivorous bats, long-term studies will have to continue to rely on access to wild colonies, with all the inherent logistic problems outlined previously in this review.

The emergence of the disastrous white-nose disease in North-America that wiped out many bat populations is a dramatic reminder that long-term studies on bats can become endangered themselves if the study species disappears, and not only because funding may cease at some point. One example of a long-term study that ended after 15 years of continuous monitoring is a Canadian study on forest-living big brown bats (*Eptesicus fuscus*). This study investigated research topics at the interface of physiology, fission–fusion behavior, colony foundation, and applied conservation (Willis and Brigham 2004; Metheny et al. 2008; Bondo et al. 2019). Another interesting example is the neo-tropical greater sac-winged bat *Saccopteryx bilineata.* The greater sac-winged bat has been studied for 50 years (M. Knörnschild et al., unpublished data), which makes it one of the best studied tropical bat species. This iconic species is well-known for its fascinating mating behavior as well as complex acoustic and olfactory communication (e.g., Nagy et al. 2007, 2012; Fernandez et al. 2021). However, because of population crashes in the respective study populations in Costa Rica and Panama (Nagy et al. 2012; M. Knörnschild et al., unpubl. data), no single marked population could be continuously monitored for more than 8–10 years, and individualized long-term data that cover the whole live of individual bats are therefore largely lacking. Fortunately, population crashes do not always mean that a long-term study needs to be terminated and instead may provide interesting insights into the drivers of population dynamics (e.g., Ward et al. 2014; Fleischer et al. 2017).

There is also hope for new long-term data becoming accessible for science: At least in Europe, many citizen scientists and conservation groups run their own banding studies on local bat populations, sometimes already since decades. By combining their long-term data on local bat populations with the statistics, population genetics, and writing skills of professional scientists, those studies can gain more international visibility and will lead to new insights into bat behavior and conservation (e.g., Schorcht et al. 2009; Reusch et al. 2019; Linton and Macdonald 2020; Stapelfeldt et al. 2022; see also Table 1). The exceptionally long-term study on greater horseshoe-bats initiated by Roger Ransome 60 years ago is a prime example of how a long-term collaboration between citizen scientists and professional scientists can lead to fantastic insights into the life of bats, ranging from demography, mating behavior, and aging to applied conservation aspects (Ransome 1989; Jones et al. 1995; Rossiter et al. 2005; Ward et al. 2014; Foley et al. 2018).

## Conclusions

Currently only few long-term field studies on individually marked bats exist that span more than 10 years, as summarized in Table 1. The existing studies have a clear regional (mostly Europe) and taxonomic bias (mostly Vespertilionid species). However, many more field studies on bats exist that have the potential to emerge into long-term studies, if the respective researchers can secure the necessary long-term funding and achieve academic tenure. I am convinced that with the further advance of miniaturized monitoring techniques, those studies will lead to new fascinating insight into the biology of bats. One notable aspect of long-term studies on bats is that they often depend on collaborations between citizen scientists, conservationists, and professional scientists. This is by no means a caveat but instead enables a fruitful exchange of knowledge among bat researchers with a different training background and facilitates the transfer of the obtained scientific results into conservation practice.
